# Variations of renal Doppler indices during the initial 24-hour predict acute kidney injury in patients with sepsis: A single-center observational case-control clinical study

**DOI:** 10.1016/j.clinsp.2024.100538

**Published:** 2025-01-26

**Authors:** Xing Chen, Wen Wu, Chao Lei, Chong Li, Zhaohui Zhang, Xingguang Qu

**Affiliations:** Department of Critical Care Medicine, The First College of Clinical Medicine Science, China Three Gorges University affiliated Yichang Central People's Hospital, Yichang, Hubei, PR China

**Keywords:** Renal Doppler index, Initial 24-hour of icu administration, Prediction, Acute kidney injury, Sepsis

## Abstract

**Background and objective:**

The aim of this retrospective observational case-control study was to examine the significance of different renal Doppler marker variations within the initial 24-hour period as potential predictors of Acute Kidney Injury (AKI) in patients with sepsis.

**Methods:**

A total of 198 sepsis patients were enrolled and categorized into two groups: the AKI group (*n* = 136) and the non-AKI group (*n* = 62). Three renal Doppler indices, Renal Resistive Index (RRI), Power Doppler Ultrasound (PDU) score and Renal Venous Stasis Index (RVSI), were measured within 6h (T0) and at 24h (T1) after ICU admission.

**Results:**

The AKI group had more hypertension patients than the non-AKI group (*p* = 0.047). The cases of the AKI group showed higher levels of CRP (*p* = 0.001), PCT (*p* < 0.001), lactate (*p* < 0.001), AST (*p* = 0.003), ALT (*p* = 0.049), total bilirubin (*p* = 0.034), BNP (*p* = 0.019) and cTnI (*p* = 0.012). The RRI at T1 was significantly higher in the AKI group (*p* = 0.037). AKI group exhibited a lower incidence of reduced RRI at T1 compared with non-AKI group (*p* < 0.001). After controlling for age, sex, and BMI through partial correlation analysis, the results indicated significant associations between SA-AKI and CVP (*r* = -0.473), SOFA score (*r* = 0.425), lactate (*r* = 0.378), and RRI reduction (*r* = -0.344) in sepsis patients. The multivariate logistic regression analysis showed that variables including CVP, SOFA score, CRP, lactate, VIS, and RRI not reduced following 24h of ICU treatment were predictive indicators for early detection of SA-AKI in sepsis patients.

**Conclusion:**

CVP, SOFA score, CRP, lactate, VIS, and RRI not reduction following 24h of ICU treatment can be utilized as predictive indicators for early detection of SA-AKI in sepsis patients.

## Highlights

• Renal Doppler marker variations within the initial 24 h in sepsis patients.
• RRI reduction after 24h of ICU treatment can be used for early detection of SA-AKI.
• Critical ultrasound for dynamic monitoring of renal perfusion.

## Introduction

Sepsis is characterized by the impairment of organ function resulting from the host's deleterious reaction to infection. Among the frequently affected organs, the kidney stands out, giving rise to Sepsis-Associated Acute Kidney Injury (SA-AKI). SA-AKI is a common complication in critically ill patients with high morbidity and mortality. Sepsis encompasses a range of different types of diseases [1]. Since the definition of Sepsis-3.0 was proposed in 2016, SA-AKI is generally defined as the type of sepsis involving the kidney, resulting in a progressive decline in renal function. Epidemiological findings showed that sepsis was associated with up to 50% of AKI, and up to 60% of sepsis patients had AKI [2]. As separate syndromes, sepsis and AKI make hosts susceptible to each other, and it is often difficult clinically to determine the exact onset timing of each syndrome. SA-AKI varies widely in the fields of disease presentation, disease progression, and treatment response, and is potentially heterogeneous. At present, there are still problems such as delayed diagnosis and lack of specific treatment in SA-AKI.

The field of renal evaluation has witnessed significant attention due to the rapid advancements in ultrasound technology. Critical ultrasound has emerged as a crucial tool for the dynamic monitoring of renal function and the early detection of AKI in critically ill patients [3,4]. The primary cause of AKI is the disruption of microcirculation hemodynamics. Monitoring of the circulation primarily focuses on the macro-circulation, whereas the critical ultrasound index can be utilized to assess the microcirculation within the kidneys. Renal Resistive Index (RRI), power Doppler Ultrasound (PDU) score and Renal Venous Stasis Index (RVSI) have been reported to be potential ultrasound markers for detecting AKI [3]. It was reported that AKI stage 3 patients had significantly higher RRI and lower PDU scores than AKI stage 0‒2 patients [5]. A study of 371 critical cases including 123 patients of persistent AKI showed that RVSI had a high sensitivity and low specificity for the differentiation of persistent AKI [6]. It is noteworthy that the majority of studies have focused on observing ultrasound indicators at a singular time point, neglecting the dynamic evaluation during early treatment. Using critical ultrasound to assess the dynamic alterations in renal blood flow following early treatment in sepsis patients could prove more advantageous in evaluating renal perfusion and predicting the risk of SA-AKI.

Thus, in this study, the authors performed a retrospective observational case-control study to analyze the variation of different ultrasound indexes among initial 24-hour Intensive Care Unit (ICU) admission and evaluate the early detective value of these variations to predict SA-AKI in sepsis patients.

## Materials and methods

### Study design, setting and location

**Fig. 1:**
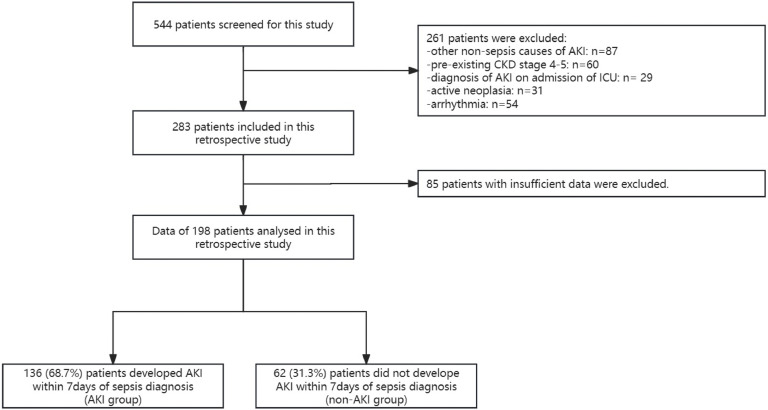
Flowchart of patient inclusion.

Following approval from the Clinical Research Ethics Committee of Yichang Central People's Hospital (2023–128–01), a retrospective observational case-control study was conducted, enrolling a total of one hundred and ninety-eight sepsis patients. These patients were admitted to Yichang Central People's Hospital between January 4th and December 30th, 2022. Utilizing the data collected within the first seven days of their sepsis diagnosis and following the guidance of KDIGO 2012, [7] the patients were categorized into two groups: the AKI group (*n* = 136) and the non-AKI group (*n* = 62) (Fig. 1). The patients who develop SA-AKI within 7-days of sepsis diagnosis were categorized in AKI group. This study followed the STROBE Statement.

### Participants

This was a retrospective observational case-control study. Patients who met the inclusion criteria were eligible: (1) Age > 18, (2) Meeting the diagnostic criteria outlined in the sepsis 3.0 guidelines [8]; (3) Matching the diagnostic criteria of AKI from KDIGO 2012 [7]. Patients with the following criteria were excluded: (1) Other factors contributing to AKI, including prior use of nephrotoxic medications, contrast-induced nephropathy, and urinary blockages; (2) Pre-existing CKD stage 4‒5 or a diagnosis of SA-AKI upon admission to ICU; (3) Pregnancy; (4) Under treatment with immunosuppressive drugs; (5) Active neoplasia; (6) Insufficiency of clinical data; (7) Coexisting disease with a high probability of death (< 6-months).

### Clinical data

The clinical data encompassed baseline demographic information such as age and sex, Body Mass Index (BMI), comorbidities, and infection sites. Additionally, blood routine, liver-kidney function, and fluid balance were documented within the initial 24 h following admission to the ICU. Furthermore, critical care scores, namely the APACHE II score and SOFA score, were recorded upon ICU admission. Vasoactive-Inotropic Score (VIS) [9] was used to evaluate the degree of vasoactive drug support for circulation.

### Renal Doppler indices measurement

Three renal Doppler indices, RRI, PDU and RVSI, were selected in the present study. The authors collected and analyzed the data of these indices within 6h (T0) and at 24h (T1) after ICU admission. The numbers of RRI reduction, PDU increase, and RVSI reduction at T1 were recorded in each group, which were used to evaluate the improvement of kidney perfusion after 24h ICU treatment. All the ultrasound tests were performed by doctors experienced in critical care ultrasound to reduce personnel error. The medical ultrasound instrument used in this study was Carnation (Wisonic, China).

The methods of measurement were followed by previous reports [10, 11, 12]. Briefly, using a 3‒5 MHz convex array probe, the long axis section of the kidney was obtained from the posterolateral side of the abdomen. Color Doppler was used to locate an interlobar artery for pulse Doppler examination, and peak systolic flow speed (VS) and minimum Diastolic flow Speed (DS) were measured [RRI = (VS-VD)/VS]. RRI was determined in three different segmental arteries (upper, middle, and lower) of both kidneys and expressed as the mean of these values.

The estimation of RVSI was conducted by assessing the peak systolic velocity minus the end-diastolic velocity, divided by the peak systolic velocity, within the renal vein. However, if the spectral waveform exhibited a monophasic or continuous pattern, the measurement of RVSI was not performed, as it necessitates the presence of multiple phases for accurate calculation. The renal blood perfusion was observed with energy Doppler, and the optimal image was saved for off-line PDU analysis according to the previous report. The right kidney was primarily evaluated for PDU due to its typically lower position and easier accessibility, while the left kidney was examined as a contingency in case the right kidney's visualization was insufficient.

### Statistical analysis

Data analysis was conducted using IBM SPSS Statistics (version 21.0, IBM, Armonk, NY, USA). Continuous data were reported as means and Standard Deviations (SDs) or medians and Interquartile Ranges (IQRs). Group comparisons were assessed using the Kruskal-Wallis test with Mann Whitney *U* test, χ^2^ test, or Fisher exact test. Spearman correlation analyses were employed to assess the relationship between indicators and SA-AKI. Partial correlation analysis was conducted to adjust for age, gender, and BMI. Logistic regression analysis was utilized to determine the relative impact of outcome variables through the calculation of unadjusted Odds Ratios (ORs). Variables that demonstrated statistical significance (*p* < 0.1) in the univariate analysis were included in the multivariate modeling. The significance level was established at 0.05.

## Results

### Baseline demographic and clinical characteristics

Initially, an examination was conducted to assess the fundamental demographic and clinical attributes of the aforementioned groups. As shown in Table 1, the AKI group had more hypertension patients than the non-AKI group (*p* = 0.047). The levels of MAP (*p* = 0.024), CVP (*p* < 0.001), and fluid output of initial 24h ICU stay (*p* < 0.001) in the AKI group were lower than those in the non-AKI group. The levels of SOFA score (*p* < 0.001), fluid balance of initial 24h ICU stay (*p* < 0.001), and vasoactive drug administration (*p* < 0.001) were higher than those in the non-AKI group.

**Table 1:** Baseline demographic and clinical characteristics.

Variables	All patients	AKI group	Non-AKI group	p-value
	*n* = 198	*n* = 136	*n* = 62	
Age (years)	65 (55, 73)	66 (55, 73.5)	64 (55, 73)	0.671
Female gender, n (%)	124 (62.6)	84 (61.8)	40 (64.5)	
BMI (Kg/m^2^)	23 (22.5, 23.3)	23.1(22.6, 23.3)	22.9 (22.5, 23.4)	0.845
History of smoking, n (%)	69 (34.8)	48 (35.3)	21 (33.9)	0.874
History of alcohol consumption, n (%)	52 (26.3)	34 (25.0)	18 (29.0)	0.603
Hypertension, n (%)	62 (31.3)	49 (36.0)	13 (21.0)	0.047
DM, n (%)	24 (12.1)	17 (12.5)	7 (11.3)	0.809
Hyperlipidaemia, n (%)	11 (5.6)	7 (5.1)	4 (6.5)	0.743
CHD, n (%)	28 (14.1)	19 (14.0)	9 (14.5)	0.919
MAP (mmHg)	85.2 ± 16.5	83.86 ± 17.49	89.03 ± 13.39	0.024
HR (bpm)	107.2 ± 23.9	108.38 ± 24.09	104.71 ± 23.66	0.317
CVP (mmHg)	5 (5, 6)	5 (4, 6)	6 (5, 7)	<0.001
APACHE II score	18 (16, 22)	18.5 (16, 22)	18 (15, 20)	0.177
SOFA score	10 (8, 13)	11 (9, 14.5)	9 (7, 9)	<0.001
Fluid infusion (mL)^a^	2578 (2097, 3352)	2578 (2012, 3332)	2579.5 (2200, 3406)	0.339
Fluid output (mL)^a^	1249 (636, 2194)	982.5 (336, 1789)	1792 (1471, 2558)	<0.001
Fluid balance (mL)^a^	1134 (539, 1953)	1497.5 (727.5, 2167.5)	727 (279, 1236)	<0.001
AKI stage (1/2/3)	76/39/21	76/39/21	–	–
Main infection site, n (%)				0.225
Lung	28 (14.1)	17 (12.5)	11 (17.7)	
Abdominal	83 (41.9)	54 (39.7)	29 (46.8)	
Skin and soft tissue	17 (8.6)	11 (8.1)	6 (9.7)	
Central nervous system	10 (5.1)	6 (4.4)	4 (6.5)	
Blood stream	36 (18.2)	31 (22.8)	5 (8.1)	
Other	24 (12.1)	17 (12.5)	7 (11.3)	
Mechanical ventilation, n (%)	106 (53.5)	75 (55.1)	31 (50.0)	0.541
Vasoactive drugs administration, n (%)	164 (82.8)	122 (89.7)	42 (67.7)	<0.001
VIS	17 (10, 34)	23 (14, 49)	11 (0, 17)	<0.001
Blood transfusions, n (%)	77 (38.9)	53 (39.0)	24 (38.7)	0.972

Note: BMI, Body Mass Index; DM, Diabetes Mellitus; CHD, Coronary Heart Disease; MAP, Mean Artery Pressure; CVP, Central Venous Pressure; CRRT, Continuous Renal Replacement Therapy; AKI, Acute Kidney Injury; HR, Heart Rate; bpm, Beat per minute; VIS, Vasoactive-Inotropic Score. Data were presented as mean ± SD or median (lower quartile, upper quartile).

a First 24 h of ICU admission.

### Laboratory characteristics at the initial 24h after ICU admission

Subsequently, an analysis of the laboratory characteristics was conducted between the two groups. The findings, as depicted in Table 2, revealed a notable disparity in platelet levels, with the AKI group exhibiting significantly lower values in comparison to the non-AKI group. Additionally, in comparison with non-AKI group, the cases of AKI group showed the higher levels of C-reactive protein CRP (*p* = 0.001), PCT (*p* < 0.001), lactate (*p* < 0.001), AST (*p* = 0.003), ALT (*p* = 0.049), total bilirubin (*p* = 0.034), BNP (*p* = 0.019) and cTnI (*p* = 0.012).

**Table 2:** Laboratory characteristics at the first 24h after ICU admission.

Variables	All patients	AKI group	Non-AKI group	p-value
	*n* = 198	*n* = 136	*n* = 62	
White Blood Cells, *10^9^	12.3 (6.9, 17.7)	12.5(6.3,18.4)	11.8 (8.2,15.4)	0.544
CRP, mg/L	163.5 (81.4, 239.9)	173.5 (116.5, 255.4)	109.9 (65.6, 196.9)	0.001
Platelet, *10^9^	91 (50, 159)	82.5 (44, 157)	123.5 (72, 165)	0.043
Procalcitonin, ng/mL	12.7 (4.6, 34.6)	15.9 (8.8, 44.1)	4.8 (1.8, 17.9)	<0.001
Serum creatinine, mmoL/L	84 (54, 134)	97.5 (46, 144)	76 (58, 94)	0.113
Lactate, mmoL/L	2.8 (1.6, 5.3)	3.7 (2.3, 6.6)	1.6 (1.2, 2.5)	<0.001
PaO2, mmHg	100.3 (73.5, 141.5)	98.2 (73.3, 139.8)	101.8 (78.5, 150.0)	0.585
PaCO2, mmHg	35.1 (29.5, 38.5)	34.7 (28.0, 38.4)	35.9 (31.5, 38.6)	0.143
AST, U/L	43.5 (23, 113)	51(27,137)	27 (21, 79)	0.003
ALT, U/L	29 (15,74)	39.5 (16, 81)	21 (14, 58)	0.049
Total bilirubin, µmoL/L	18.4 (11.4, 35.8)	20.5(12, 42.4)	14.9 (10.1, 28.9)	0.034
BNP, ng/L	100.4 (56.4, 224.7)	112.6 (58.8, 245.7)	80.3 (45.4, 156,7)	0.019
Cardiac troponin I, µg/L	0.01 (0.01, 0.04)	0.01 (0.01, 0.07)	0.01 (0.01, 0.01)	0.012

Note: AST, Aspartate Aminotransferase; ALT, Alanine Aminotransferase; BNP, Brain Natriuretic Peptide. Data were presented as median (lower quartile, upper quartile).

### Ultrasound index

Subsequently, the authors conducted an analysis of the ultrasound indexes between the two groups. As shown in Table 3, the RRI at T1 was found to be significantly higher in the AKI group compared to the non-AKI group (*p* = 0.037). AKI group exhibited a lower incidence of reduced RRI at T1 compared with non-AKI group (*p* < 0.001).

**Table 3:** Ultrasound indexes in AKI group and non-AKI group.

Variables	All patients	AKI group	Non-AKI group	p-value
	*n* = 198	*n* = 136	*n* = 62	
RRI				
T0	0.69 (0.66, 0.72)	0.69 (0.66, 0.72)	0.70 (0.65, 0.73)	0.183
T1	0.68 (0.64, 0.73)	0.69 (0.65, 0.73)	0.67 (0.62, 0.72)	0.037
Number of RRI reduction, n (%)	96 (48.5)	53 (39.0)	43 (69.4)	<0.001
PDU score				
T0	2 (2, 3)	2 (2, 3)	2 (2, 3)	0.467
T1	2 (2, 3)	2 (2, 3)	2 (2, 3)	0.513
Number of PDU increase, n (%)	29 (14.6)	16 (11.8)	13 (21.0)	0.128
RVSI				
T0^a^	0.43 ± 0.07	0.43 ± 0.08	0.42 ± 0.07	0.696
T1^b^	0.43 ± 0.07	0.44 ± 0.08	0.42 ± 0.07	0.160
Number of RVSI reduction, n (%)^c^	29 (43.3)	17 (38.6)	12 (52.2)	0.312

Note: RRI, Renal Resistive Index; PDU, Lower Semiquantitative Power Doppler; RVSI, Renal Venous Stasis Index; T0, Within 6h of ICU admission; T1, At 24h after ICU admission. Data were presented as mean ± SD or median (lower quartile, upper quartile).

a The number of biphasic renal vein flow pattern were 66 (48.5%) and 29 (46.8%) in AKI group and non-AKI group, respectively.

b The number of biphasic renal vein flow pattern were 57 (51.9%) and 28 (45.2%) in AKI group and non-AKI group, respectively.

c The data number were 44 (32.4%) and 23 (37.1%) in AKI group and non-AKI group, respectively.

### Correlation analysis

Then, the authors conducted a Spearman correlation analysis to assess the association between each marker and SA-AKI. As depicted in Table 4, the results revealed that lactate (*r* = 0.487), SOFA score (*r* = 0.441), RRI reduction (*r* = −0.382), and CVP (*r* = −0.385) were the first four indicators related to AKI development in sepsis patient. Furthermore, following the adjustment of age, sex, and BMI via partial correlation analysis, these four markers, CVP (*r* = −0.473), SOFA score (*r* = 0.425), lactate (*r* = 0.378), RRI reduction (*r* = −0.344) were still significantly associated with SA-AKI in sepsis patients.

**Table 4:** Correlation analysis.

	*r*	*r*_adjusted_ age	*r*_adjusted_ age and sex	*r*_adjusted_ age, sex, and BMI
**Hypertension**	0.151^▲^	0.212	0.211	0.194
**MAP**	−0.172^▲^	−0.081	−0.078	−0.048
**CVP**	−0.385▲▲	−0.473▲▲▲	−0.479▲▲▲	−0.473▲▲▲
**SOFA score**	0.441▲▲	0.452▲▲▲	0.451▲▲▲	0.425▲▲▲
**Fluid balance**	0.292▲▲	0.204	0.203	0.168
**Vasoactive drugs**	0.270▲▲	0.197	0.197	0.157
**VIS**	0.340▲▲▲	0.355▲▲▲	0355▲▲▲	0.338▲▲▲
**CRP**	0.248▲▲	0.340▲▲	0.340▲▲	0.339▲▲
**PCT**	0.345▲▲	0.142	0.147	0.095
**Platelet**	−0.145^▲^	−0.039	−0.035	0.104^a^
**Lactate**	0.487▲▲	0.399▲▲	0.399▲▲	0.378▲▲
**AST**	0.209▲▲	0.149	0.147	0.115
**ALT**	0.141^▲^	0.150	0.148	0.114
**Total bilirubin**	0.151^▲^	0.250^▲^	0.251^▲^	0.248^▲^
**BNP**	0.167^▲^	0.105	0.101	0.114
**cTnI**	0.178^▲^	0.085	0.080	0.092
**RRI**				
T0	−0.095	0.002	0.002	−0.007
T1	0.149^▲^	0.253^▲^	0.252^▲^	0.249^▲^
RRI reduction	−0.382▲▲▲	−0.360▲▲▲	−0.358▲▲▲	−0.344▲▲▲
**PDU score**				
T0	0.052	0.015	0.015	0.025
T1	−0.047	−0.113	−0.114	−0.115
PDU increase	0.121	0.109	0.110	0.131
**RVSI**				
T0^a^	0.035	0.042	0.041	0.007
T1^b^	0.154	0.180	0.181	0.214
**RVSI reduction**^c^	−0.130	−0.136	−0.140	−0.187

Note: AST, Aspartate Aminotransferase; ALT, Alanine Aminotransferase; BNP, Brain Natriuretic Peptide. BMI, Body Mass Index; MAP, Mean Artery Pressure; CVP, Central Venous Pressure; VIS, Vasoactive-Inotropic Score; RRI, Renal Resistive Index; PDU, Lower Semiquantitative Power Doppler; RVSI, Renal Venous Stasis Index. T0, Within 6h of ICU admission; T1, At 24h after ICU admission.

a The number of biphasic IRVF pattern were 66 (48.5%) and 29 (46.8%) in AKI group and non-AKI group, respectively.

b The number of biphasic IRVF pattern were 57 (51.9%) and 28 (45.2%) in AKI group and non-AKI group, respectively.

c The data number were 44 (32.4%) and 23 (37.1%) in AKI group and non-AKI group, respectively. ^▲^*p* < 0.05.

▲▲ *p* < 0.01.

▲▲▲ *p* < 0.001.

### Logistic regression

Next, to assess the risk factors associated with SA-AKI in sepsis patients, the authors conducted univariate and multivariate logistic regression analyses. The outcomes of the univariate logistic regression, as presented in Table 5, demonstrated that. MAP, CVP, SOFA score, vasoactive drug treatment, VIS, CRP, PCT, lactate, BNP, RRI at T1, and RRI reduction were associated with AKI in sepsis cases. After adjusting for potential confounding factors such as age and sex, a multivariate logistic regression analysis showed that variables including CVP, SOFA score, CRP, lactate, VIS, and RRI not reduction following 24h of ICU treatment could be utilized as predictive indicators for early detecting of SA-AKI in sepsis patients.

**Table 5:** Logistic regression.

	Univariate analysis		Multivariate analysis	
	OR	p-value	OR	p-value
Age	1.005	0.651		
Sex (Male)	1.126	0.711		
Hypertension	2.123	0.036	2.443	0.079
MAP	0.981	0.042	1.004	0.782
CVP	0.508	<0.001	0.424	<0.001
SOFA score	1.461	<0.001	1.270	0.021
Positive fluid balance	2.140	0.119		
Vasoactive drugs	4.150	<0.001	1.544	0.485
VIS	1.073	<0.001	1.056	0.020
CRP	1.006	0.001	1.008	0.004
PCT	1.015	0.005	1.004	0.505
Platelet	0.998	0.212		
Lactate	1.746	<0.001	1.269	0.036
AST	1.000	0.913		
ALT	1.000	0.694		
Total bilirubin	1.007	0.131		
BNP	1.003	0.021	1.003	0.140
cTnI	1.239	0.614		
RRI at T0	0.007	0.201		
RRI at T1	384.6	0.025	1.440	0.937
RRI reduction at T1	0.282	<0.001	0.389	0.037
PDU at T0	1.180	0.522		
PDU at T1	0.817	0.512		
PDU increase at T1	0.503	0.093		
RVSI at T0^a^	3.307	0.692		
RVSI at T1^b^	90.18	0.160		
RVSI reduction at T1^c^	0.577	0.290		

Note: AST, Aspartate Aminotransferase; ALT, Alanine Aminotransferase; BNP, Brain Natriuretic Peptide. BMI, Body Mass Index; MAP, Mean Artery Pressure; CVP, Central Venous Pressure; VIS, Vasoactive-Inotropic Score; RRI, Renal Resistive Index; PDU, Lower Semiquantitative Power Doppler; RVSI, Renal Venous Stasis Index. T0, Within 6h of ICU admission; T1, At 24h after ICU admission.

a The number of biphasic IRVF pattern were 66 (48.5%) and 29 (46.8%) in AKI group and non-AKI group, respectively.

b The number of biphasic IRVF pattern were 57 (51.9%) and 28 (45.2%) in AKI group and non-AKI group, respectively.

c The data number were 44 (32.4%) and 23 (37.1%) in AKI group and non-AKI group, respectively.

## Discussion

Sepsis is characterized by organ dysfunction, and it has been observed that approximately 40% to 50% of sepsis patients may experience AKI, which has been found to be associated with a significant 6‒8 fold rise in mortality rates and an increased risk of developing CKD [2,13,14]. The physiological mechanism of SA-AKI includes inflammation, impairment of microcirculatory blood flow and the cellular bioadaptive response to tissue injury [1,2,15]. The change of renal blood perfusion, including renal macrocirculation and microcirculation, is the key process in sepsis-induced AKI [16]. CT, MRI, and PET imaging techniques have the capability to assess the renal blood perfusion. However, their clinical utility is constrained by factors such as the high cost, long examination time, and potential toxicity of contrast agents, thereby limiting their widespread application in clinical settings [17]. Renal ultrasound serves as a widely used imaging method for the evaluation of renal blood perfusion with few side effects and no renal toxicity. Thus, in this study, the authors performed a retrospective study of 198 cases to analyze the variation of different renal ultrasound blood flow indexes during the initial 24 h of ICU admission. Our results indicated that variables including CVP, SOFA score, CRP, lactate, VIS, and RRI not reduced following 24h of ICU treatment could be utilized as predictive indicators for early detection of SA-AKI in sepsis patients.

In this study, the authors selected three renal blood flow ultrasound indexes including RRI, PDU and RVSI. These results showed that the T1 levels of RRI were significantly elevated in the AKI group compared to the non-AKI group. Furthermore, the AKI group exhibited a lower incidence of reduced RRI at T1 in comparison to the non-AKI group. Correlation analysis revealed that RRI reduction at T1 was negatively associated with SA-AKI. Following the adjustment of age, sex, and BMI via partial correlation analysis, reduced RRI at T1 also presented a negative relation to SA-AKI development. RRI at T0, PDU, and RVIS did not exhibit the aforementioned characteristics. RRI reflects the blood flow resistance of renal vessels and is widely used to reflect the status of renal parenchyma. RRI is a semi-quantitative index calculated by dividing the difference between peak systolic and end-diastolic velocity by the peak systolic velocity. Ultrasonic measurements of renal arcuate or interlobar arteries are used to determine velocity. According to the previous reports, normal RRI ranges from 0.47 to 0.70 with less than 5%‒8% difference between two kidneys [18]. These results showed that the median RRI at T1 were 0.69 and 0.67 in AKI cases and non-AKI cases, respectively. It was reported that RRI ≥0.7 was an independent risk factor for progression to irreversible renal failure in CKD patients [19]. Wiersema's study enrolled 371 critical cases and showed that RRI did not differ between patients with and those without persistent AKI which was defined as any AKI that persisted more than three days. RRI had a moderate specificity and low sensitivity for persistent AKI [6]. There were around one-third surgery cases in Wiersema's study which means a considerable proportion were not sepsis patients. The RRI of Wiersema's study was only measured at ICU admission. These factors may explain the different results between Wiersema's study and this study. Song's study suggested that high CVP and RRI at admission were independent factors for SA-AKI [20]. However, the present results indicated AKI group had a lower CVP level at ICU admission and RRI reduced following 24-hour ICU treatment, but not RRI at ICU admission was more related to SA-AKI. The perfusion of the kidneys is contingent upon the disparity between MAP and CVP. An elevation in MAP or a reduction in CVP will result in an augmentation of renal perfusion pressure. The present results showed AKI group had lower MAP levels and higher CVP levels compared with the non-AKI group at ICU admission. This means the patients of the AKI group were under lower renal perfusion pressure conditions. The correlation and regression analysis results indicated that CVP was more related to SA-AKI than MAP. Furthermore, the above studies did not consider the interference of ICU treatment on renal perfusion. In this study, the authors analyzed the variation of RRI among the first 24 h of ICU admission, we found that RRI reduced following 24h ICU treatment, but not RRI at ICU admission was more related to SA-AKI. The authors believe that the dynamic trend of indicators is more reliable than the single time point data. It is noted that the AKI group presented more patients with hypertension, which might be a bias for RRI value [21]. In the present study, the authors tried to eliminate the bias facts. First, the authors used univariate and multivariate logistic regression analyses. The regression results indicated hypertension was not the risk factor for SA-AKI. Second, we set and analyzed the value of RRI reduction following 24h of ICU treatment, which indicated the changes in RRI. Hypertension may influence the value of RRI, but it cannot affect the changes of RRI following CRRT treatment. Thus, based on those, the bias of hypertension to RRI is limited.

The PDU method utilizes the energy of red blood cells within the bloodstream to present a blood flow signal, which remains unaffected by the direction of blood flow or the angle between blood flow and the sound beam [22]. The PDU score has been found to offer a more straightforward and convenient way to assess renal perfusion compared with RRI, while also providing a comparable evaluation of renal function [23]. The results did not find significant differences of PDU between SA-AKI cases and non-SA-AKI cases. Yu's previous study reported that there was no significant difference in the PDU scores within 7 days after ICU admission between the SA-AKI and non-SA-AKI patients [24]. Most patients in Yu's study scored 2 points which were consistent with our results. Some limitations of PDU may contribute to this negative results. First, PDU score is a kind of semi-quantitative detection method, that introduces subjective bias. Second, the rating of PDU result can be affected by the soft tissue flash artifact caused by respiratory movement. Third, the ICU population often includes patients with obesity, abdominal distention, bowel dilation, or limited postural movement, all of which can potentially impact the accuracy of the PDU score.

The RVSI serves as a crucial metric for assessing renal afterload [25]. Within the venous component of renal blood circulation, flow resistance is minimal, with compliance emerging as the primary factor influencing renal afterload. Consequently, RVSI offers a direct measure of renal vein compliance, thereby enabling the evaluation of renal vein congestion [26,27]. The calculation of RVSI is similar to RRI. Wiersema's study enrolled 371 critical cases and revealed that RVSI exhibited a nearly 90% false positive rate for predicting AKI which means RVSI could not independently predict AKI risk [6]. The present results were consistent with Wiersema's study. The authors also found that there were no differences on T0 or T1 RVSI between AKI and non-AKI groups. Furthermore, the authors found only half cases presented a biphasic renal vein flow pattern, which indicated RVSI could not be measured in nearly half of sepsis patients.

The present study's data indicated that lower SOFA and reduced need for vasopressors were more frequently observed in sepsis patients who were not developed to SA-AKI. The conclusion of this study suggested SOFA score and VIS were also the predictive indicators for early detection of SA-AKI in sepsis patients. SOFA score has been reported as an early identification marker for SA-AKI [28,29]. VIS is the index to evaluate the degree of vasoactive drug support for circulation. It is reported that, in children with severe sepsis, the degree of hemodynamic support as measured by the VIS may identify patients at increased risk of developing SA-AKI [30].

This study is subject to certain limitations. First, the retrospective and single-center design of the study introduces a potential bias risk. Therefore, it is imperative to conduct a future prospective study with a larger population to validate the findings and conclusions of this research. Second, hemodynamic status may affect RRI and PDU scores. Third, some cases of this study were CKD stages 1 to 3. CKD may damage the renal vascular system and lead to elevated RRI.

In summary, this study included a total of 198 patients diagnosed with sepsis, out of which 136 exhibited SA-AKI. The present findings indicate that the measurement of CVP, SOFA score, CRP levels, lactate levels, and RRI reduction after 24 h of ICU treatment can serve as valuable predictive indicators for the early detection of SA-AKI in sepsis patients. Hypertension patients had lower basal renal cortical perfusion and impaired renal cortical flow.

## Consent for publication

Not applicable.

## Ethics approval and consent to participate

Clinical Research Ethics Committee of Yichang Central People's Hospital (2023–128–01). All methods were carried out in accordance with relevant guidelines and regulations. As it was a retrospective study, the Clinical Research Ethics Committee of Yichang Central People's Hospital waived the need for informed consent in the study.

## Authors’ contributions

XGQ and XC conceived the research. XC, WW, ChaoL, and ChongL collected and analyzed the clinical data. XC designed the study. XC drafted the manuscript. ZHZ and XGQ reviewed the final manuscript. All authors read and approved the final manuscript.

## Funding

Not applicable.

## Declaration of competing interest

The authors declare no conflicts of interest.

## Data Availability

The datasets used and/or analyzed during the current study available from the corresponding author on reasonable request.
